# 宏基因组二代测序在异基因造血干细胞移植后耶氏肺孢子菌肺炎诊断中的意义

**DOI:** 10.3760/cma.j.cn121090-20230928-00147

**Published:** 2024-01

**Authors:** 蓉 符, 韧 林, 志平 范, 芬 黄, 娜 许, 丽 宣, 怡霏 黄, 慧 刘, 珂 赵, 治香 王, 玲 蒋, 敏 戴, 竞 孙, 启发 刘

**Affiliations:** 南方医科大学南方医院血液科，南方医科大学血液病研究所，广东省血液系统疾病临床医学研究中心，广州 510515 Department of Hematology, Nanfang Hospital, Southern Medical University, Clinical Medical Research Center of Hematological Diseases of Guangdong Province, Guangzhou 510515, China

**Keywords:** 耶氏肺孢子菌肺炎, 异基因造血干细胞移植, 宏基因组二代测序, Pneumocystis jirovecii pneumonia, Allogeneic hematopoietic stem cell transplantation, Metagenomic next-generation sequencing

## Abstract

**目的:**

评估宏基因组二代测序（mNGS）对异基因造血干细胞移植（allo-HSCT）患者耶氏肺孢子菌肺炎（PJP）的诊断价值。

**方法:**

纳入2019年6月至2023年8月南方医科大学南方医院血液科allo-HSCT后疑似肺感染并接受肺泡灌洗液病原学检测的98例患者，比较mNGS与常规方法、实时定量PCR（RQ-PCR）对PJP的诊断效能。

**结果:**

12例患者最终诊断为PJP（确诊11例、临床诊断1例）。确诊患者中1例为常规方法和RQ-PCR检测均阳性，10例仅RQ-PCR阳性。12例患者mNGS均检出耶氏肺孢子菌。mNGS诊断PJP的敏感性为100％，高于常规方法（8.3％，*P*＝0.001），与RQ-PCR（91.6％）相当（*P*＝1.000）。75％的PJP患者为肺混合感染，合并EB病毒和巨细胞病毒最为常见。mNGS检出混合感染8例，RQ-PCR检出5例，常规方法未检出（*P*＝0.008）。

**结论:**

mNGS在allo-HSCT患者PJP诊断中具有良好的敏感性，在混合感染病原检测方面具有优势，可作为常规检测方法及RQ-PCR的有效补充。

## Abstract (en)

**Objective:**

To investigate the value of metagenomic next-generation sequencing（mNGS）in the diagnosis of Pneumocystis jirovecii pneumonia（PJP）in patients undergoing allogeneic hematopoietic stem cell transplantation（allo-HSCT）.

**Methods:**

The data of 98 patients with suspected pulmonary infection after allo-HSCT who underwent pathogen detection from bronchoalveolar lavage fluid between June 2016 and August 2023 at Nanfang Hospital were analyzed. The diagnostic performance of mNGS, conventional methods, and real-time quantitative polymerase chain reaction（qPCR）for PJP were compared.

**Results:**

A total of 12 patients were diagnosed with PJP, including 11 with a proven diagnosis and 1 with a probable diagnosis. Among the patients with a proven diagnosis, 1 was positive by both conventional methods and qPCR, and 10 were positive by qPCR only. Pneumocystis jirovecii was detected by mNGS in all 12 patients. The diagnostic sensitivity of mNGS for PJP was 100%, which was greater than that of conventional methods（8.3%, *P*＝0.001）and similar to that of qPCR（91.6%, *P*＝1.000）. A total of 75% of the patients developed mixed pulmonary infections, and cytomegalovirus and Epstein-Barr virus were the most common pathogens. Mixed infection was detected in eight patients by mNGS and in five patients by qPCR, but not by conventional methods（*P*＝0.008）.

**Conclusion:**

mNGS had good sensitivity for diagnosing PJP after allo-HSCT and was advantageous for detecting mixed infectious pathogens; therefore, mNGS might be an effective supplement to regular detection methods and qPCR.

耶氏肺孢子菌是引起人类耶氏肺孢子菌肺炎（PJP）的机会性致病菌[1]，是异基因造血干细胞移植（allo-HSCT）后的严重感染并发症[2]–[3]。尽管allo-HSCT后患者接受磺胺类药物预防，仍有部分患者会发生PJP，且病情发展迅速，病死率高[4]–[5]。肺孢子菌极难在体外培养，痰、肺泡灌洗液（BALF）、肺组织活检等标本直接镜检或特异性染色发现肺孢子菌是诊断PJP的金标准[6]，但这些方法敏感性低，阴性结果不能排除PJP诊断[7]。目前，allo-HSCT患者PJP诊断常基于临床症状、影像学表现及治疗反应[8]。近年来，实时定量PCR（RQ-PCR）已应用于肺孢子菌临床检测且具有良好的敏感性和特异性[9]，第五届欧洲白血病感染会（ECIL-5）推荐其作为PJP的常规诊断方法[10]。但PCR检测肺孢子菌的诊断阈值目前还没有统一标准且需要在采样前预判病原体以免遗漏送检[11]。宏基因组二代测序（mNGS）通过直接对样本中的全部核酸片段进行无偏倚测序，理论上能快速识别包括病毒、细菌、真菌和寄生虫在内的所有病原体[12]，已逐渐应用于感染病原学检测[13]–[15]。在免疫功能低下人群中的研究表明，mNGS可能是诊断PJP的一个很有前景的检测方法[16]–[19]。然而，mNGS对allo-HSCT患者PJP诊断的研究较少，其诊断效能尚不清楚。因此，我们回顾性分析本中心接受BALF病原学检查并诊断为PJP的allo-HSCT患者，评估mNGS对allo-HSCT后PJP的诊断效能。

## 病例与方法

一、研究对象

纳入2019年6月至2023年8月在南方医科大学南方医院血液科因疑似肺感染住院的allo-HSCT后患者。符合以下标准的患者纳入分析：①出现疑似肺感染症状即发热、咳嗽或呼吸困难；②新发胸部CT影像学异常；③经验性抗生素治疗72 h无效。所有纳入分析的患者均接受纤维支气管镜检查，采集BALF标本行常规方法、RQ-PCR以及mNGS检测病原体。

PJP诊断基于临床症状、胸部影像学、微生物学检查和治疗反应综合判定[10],[20]。BALF染色镜检找到肺孢子菌包囊或滋养体，或肺孢子菌RQ-PCR检测结果阳性视为检出耶氏肺孢子菌，即为确诊。若未找到病原学证据，但结合临床症状、胸部影像学以及治疗反应综合判断为PJP，即为临床诊断。

二、实验室检测方法

常规检测方法包括BALF进行一般细菌真菌涂片、镜检染色（革兰染色、抗酸染色、墨汁染色、六亚甲基四胺银染色）、细菌真菌培养、1,3-β-D葡聚糖试验（G试验）、半乳甘露聚糖试验（GM试验）。采用RQ-PCR检测肺孢子菌，若循环阈值（CT）<40，则为肺孢子菌RQ-PCR阳性。同时采用RQ-PCR检测病毒和曲霉菌。

三、mNGS检测方法

无菌留取10 ml BALF进行mNGS检测，样本加入到NGSmaster™自动化工作站中进行自动化核酸提取、逆转录（仅RNA）、核酸片段化、末端补平、末端腺苷化（3′端加单碱基A）、测序接头（adapter）连接、纯化后形成测序文库。将文库通过荧光定量PCR仪进行文库定量后，使用Illumina Nextseq™高通量测序平台对文库进行鸟枪法测序。每个文库预期读取产生2000万条单端50 bp的序列数据。对读取的文库序列数据进行生物信息学分析，过滤其中的人类基因组序列数据（GRCh38.p13），将其余序列数据与微生物参考数据库（NCBI GenBank和内部策划的微生物基因组数据）进行比对，以确定微生物物种和相对丰度。每一轮mNGS检测都会加入一个阴性对照（血浆游离核酸及片段化的人类基因组DNA混合物）和一个阳性对照（包含灭活细菌、真菌和假病毒颗粒的混合物）。由于真菌其提取DNA的生物量较低，其检出的特异序列数相对其他微生物较少，因此mNGS检出其特异性序列数是其他真菌的5倍以上，则耶氏肺孢子菌被认为是阳性[21]。

四、统计学处理

采用SPSS统计软件26.0进行数据分析。连续变量用“中位数（范围）”表示，分类变量用百分比表示。以最终诊断作为标准，采用卡方检验或Fisher精确检验比较常规方法、RQ-PCR和mNGS诊断PJP的敏感性、特异性、阳性预测值（PPV）和阴性预测值（NPV）。*P*值均为双侧，显著性水平α＝0.05。

## 结果

一、PJP患者临床特征

共98例患者纳入分析，其中12例最终诊断为PJP（11例确诊，1例临床诊断）。其中男10例，女2例，中位年龄为46（19～68）岁，2例合并慢性移植物抗宿主病（GVHD），发生PJP的中位时间为allo-HSCT后257（43～992）d。12例PJP患者的临床资料见表1。

**表1:** 12例耶氏肺孢子菌肺炎（PJP）患者的临床特征及实验室检查

特征	结果
中位年龄［岁，*M*（范围）］	46（19~68）
性别［例（%］	
男	10（83.3）
女	2（16.7）
临床症状［例（%）］	
发热	9（75.0）
咳嗽咳痰	6（50.0）
呼吸困难	9（75.0）
肺CT［例（%）］	
磨玻璃样密度影	7（58.3）
斑片状或条索密度影	8（66.7）
小结节	6（50.0）
实验室检查［*M*（范围）］	
白细胞（×10^9^/L）	6.98（4.71~11.11）
中性粒细胞（×10^9^/L）	4.37（2.00~8.98）
淋巴细胞（×10^9^/L）	1.15（0.66~4.01）
超敏C反应蛋白（mg/L）	58.10（1.39~99.21）
乳酸脱氢酶（U/L）	258（124~685）
原发病［例（%）］	
急性髓系白血病	6（50.0）
急性淋巴细胞白血病	3（25.0）
骨髓增生异常综合征	3（25.0）
供者类型［例（%）］	
同胞	12（100.0）
无关	0（0）
供者HLA相合程度［例（%）］	
全相合	7（58.3）
非全相合	5（41.7）
造血干细胞来源［例（%）］	
外周血干细胞	4（33.3）
骨髓+外周血干细胞	8（66.7）
预处理方案［例（%）］	
TBI+Cy	3（25.0）
Bu+Cy	9（75.0）
GVHD［例（%）］	
急性	0（0）
慢性	2（16.7）
移植后PJP发生的中位时间［d，*M*（范围）］	257（43~992）

注 TBI：全身放射治疗；Cy：环磷酰胺；Bu：白消安

二、PJP患者BALF病原学结果

12例PJP患者的常规方法、RQ-PCR和mNGS检测结果见表2。11例确诊患者中，1例为常规方法和RQ-PCR检测均为阳性，10例仅RQ-PCR阳性。1例临床诊断患者连续2次G试验阳性（>60 ng/L），结合临床特征及治疗反应，最终诊断为PJP。12例患者mNGS均检测出耶氏肺孢子菌，序列数为8-1481。12例PJP患者中9例合并其他病原体阳性，EB病毒和巨细胞病毒最为常见，其次为溶血葡萄球菌、鲍曼不动杆菌、肺炎链球菌、曲霉菌。mNGS检出混合感染8例，RQ-PCR检出混合感染5例，常规方法未检出混合感染（表2），mNGS对于混合感染的检出率明显高于常规方法（*P*＝0.008）。

**表2:** 12例耶氏肺孢子菌肺炎（PJP）患者病原体检测结果、治疗及转归

例号	常规方法	mNGS	RQ-PCR	治疗	转归
1	阴性	耶氏肺孢子菌序列数15，CMV序列数60	肺孢子菌	卡泊芬净	生存
2	阴性	耶氏肺孢子菌序列数76，溶血葡萄球菌序列数57	肺孢子菌	TMP-SMZ+卡泊芬净	生存
3	阴性	耶氏肺孢子菌序列数20，EBV序列数63，CMV序列数45	肺孢子菌，EBV，CMV	TMP-SMZ	生存
4	阴性	耶氏肺孢子菌序列数1481	肺孢子菌	TMP-SMZ	生存
5	阴性	耶氏肺孢子菌序列数430	肺孢子菌	TMP-SMZ+卡泊芬净	生存
6	阴性	耶氏肺孢子菌序列数23，CMV序列数40	肺孢子菌，CMV	TMP-SMZ+卡泊芬净	生存
7	阴性	耶氏肺孢子菌序列数69	肺孢子菌，曲霉菌	TMP-SMZ	生存
8	G试验连续2次阳性	耶氏肺孢子菌序列数8，鲍曼不动杆菌序列数642，EBV序列数9	阴性	TMP-SMZ+卡泊芬净	生存
9	阴性	耶氏肺孢子菌序列数447	肺孢子菌	TMP-SMZ+卡泊芬净	生存
10	六亚甲基四胺银染色阳性	耶氏肺孢子菌序列数9，EBV序列数11	肺孢子菌	TMP-SMZ	生存
11	阴性	耶氏肺孢子菌序列数94，EBV序列数7	肺孢子菌，EBV	TMP-SMZ+卡泊芬净	生存
12	阴性	耶氏肺孢子菌序列数13，EBV序列数6494，肺炎链球菌序列数66	肺孢子菌，EBV	TMP-SMZ	生存

注 mNGS：宏基因组二代测序；RQ-PCR：实时定量-聚合酶链反应；CMV：巨细胞病毒；EBV：EB病毒；TMP：甲氧苄啶；SMZ：磺胺甲噁唑

三、mNGS与常规方法、RQ-PCR检测对PJP诊断效能比较

mNGS诊断PJP的敏感性显著高于常规方法［100.0％（95％*CI* 69.9％～100.0％）对8.3％（95％*CI* 0.4％～40.2％），*P*＝0.001］，与RQ-PCR敏感性［91.6％（95％*CI* 59.8％～99.6％）］相当（*P*＝1.000，表3）。mNGS诊断PJP的特异性为98.8％（95％ *CI* 92.8％～99.9％），与常规方法［100.0％（95％*CI*　94.7％～100.0％）］及RQ-PCR［100.0％（95％*CI* 94.7％～100.0％）］比较，差异均无统计学意义（*P*值均为1.000，表3）。mNGS诊断PJP的PPV、NPV分别为92.3％（95％*CI* 62.1％～99.6％）、100.0％（95％*CI* 94.6％～100.0％），与RQ-PCR比较差异无统计学意义（PPV：*P*＝0.347；NPV：*P*＝0.322，表3）。mNGS的PPV与常规方法相似（*P*＝0.773），但NPV高于常规方法（*P*＝0.001，表3）。

**表3:** mNGS与常规方法、RQ-PCR对耶氏肺孢子菌肺炎（PJP）患者的诊断效能比较

方法	敏感性（95% *CI*）	特异性（95% *CI*）	PPV（95% *CI*）	NPV（95% *CI*）
mNGS	100.0%（69.9%~100.0%）	98.8%（92.8%~99.9%）	92.3%（62.1%~99.6%）	100.0%（94.6%~100.0%）
常规方法	8.3%（0.4%~40.2%）^a^	100.0%（94.7%~100.0%）^b^	100.0%（5.5%~100.0%）^b^	88.7%（80.2%~94.5%）^a^
RQ-PCR	91.6%（59.8%~99.6%）^b^	100.0%（94.7%~100.0%）^b^	100.0%（67.9%~100.0%）^b^	98.9%（92.9%~99.9%）^b^

注 mNGS：宏基因组二代测序；RQ-PCR：实时定量-聚合酶链反应；PPV：阳性预测值；NPV：阴性预测值。与mNGS比较，^a^*P*<0.05；与mNGS比较，^b^*P*>0.05

四、PJP患者的治疗及预后

3例PJP患者在送检mNGS前经验性抗感染用药已覆盖PJP，9例患者后续根据mNGS结果调整抗耶氏肺孢子菌感染方案。最终1例患者接受卡泊芬净单药治疗，5例甲氧苄啶/磺胺甲噁唑（TMP-SMZ）单药治疗，6例TMP-SMZ联合卡泊芬净。12例患者均接受糖皮质激素治疗。12例患者均治愈（表2）。

## 讨论

本回顾性研究比较了BALF标本mNGS、常规方法、RQ-RCR检测在allo-HSCT后PJP患者中的诊断效能，我们发现BALF mNGS对allo-HSCT后PJP的诊断具有良好的敏感性和特异性。同时我们发现PJP患者多为混合感染，mNGS有利于混合感染中共致病菌的诊断与鉴别。

allo-HSCT目前仍然是治愈恶性血液系统疾病、某些遗传病和自身免疫性疾病的唯一手段[22]。在实体器官移植、造血干细胞移植、恶性肿瘤以及使用免疫抑制药物治疗的自身免疫病患者等非人类免疫缺陷病毒（HIV）感染的免疫缺陷群体中，耶氏肺孢子菌感染的发病率呈上升趋势[23]–[24]。既往肺孢子菌的检测主要依赖直接镜检或特异性染色，其敏感性低，而G试验结果缺乏病原特异性，故往往仅基于临床表现和影像学表现作出临床诊断[10],[25]，容易造成诊断不及时或漏诊。PCR在免疫功能低下人群中对肺孢子菌感染具有良好的诊断效能[26]，但PCR单次检测通常仅聚焦于一种致病菌，在混合感染中的价值有限，且缺乏统一的诊断阈值[11]。

mNGS具有无偏倚、快速、高灵敏度等特点，在肺感染中应用越来越广泛，尤其适用于常规诊断方法无法识别的病原体[27]。Jiang等[28]在非HIV感染的免疫抑制患者合并PJP的研究结果显示，mNGS的敏感度为100.0％，特异性为96.3％。Chen等[29]在危重儿科患者的研究结果亦表明mNGS诊断PJP的敏感性（100.0％）和特异性（96.7％）均高于常规方法。本研究中mNGS对诊断PJP的敏感性为100.0％，明显高于常规检测方法，与文献[12],[28]–[29]报告的结果相符。以往研究报道RQ-PCR诊断PJP特异性可达到90％以上[26]。本研究mNGS诊断PJP的特异性为98.8％，与RQ-PCR相当。与其他微生物学检测方法相比，mNGS的另一个明显优势是病原体鉴定的广谱性。文献显示PJP患者常见肺混合感染[30]。在本研究中，PJP患者混合感染占75％，mNGS有助于混合感染中其他共病原体的诊断与鉴别，能为混合感染提供更全面的抗感染治疗依据。

在使用磺胺类药物预防之前，PJP在造血干细胞移植患者中的发生率为5％～16％[5]，磺胺类药物常规预防使PJP的发生率降至1％～6％[4]。allo-HSCT患者PJP多发生于移植后1年内，Xu等[31]研究发现allo-HSCT后PJP中位发生时间为189 d；沈再红等[2]报告的PJP中位发生时间为221 d。本组病例移植后并发PJP的中位时间为257（43～992）d。这些数据提示移植后患者或需规律预防PJP至移植后1年。

TMP-SMZ是PJP的一线治疗药物[25]。对于HIV和器官移植后的重症PJP患者，共识均推荐糖皮质激素作为辅助治疗，可降低其死亡率[32]–[33]，但allo-HSCT后PJP患者接受糖皮质激素治疗是否能改善预后尚不明确。本组患者在诊断后采用TMP-SMZ或联合卡泊芬净抗PJP治疗，同时予糖皮质激素辅助治疗，12例患者均治疗有效并存活。

本研究结果显示，mNGS在allo-HSCT患者PJP诊断中具有良好的敏感性，且在混合感染病原检测方面具有优势，可作为常规检测方法及RQ-PCR的有效补充。
